# Coverage Retention and Plan Switching Following Switches From a Zero- to a Positive-Premium Plan

**DOI:** 10.1001/jamahealthforum.2025.1424

**Published:** 2025-05-23

**Authors:** Coleman Drake, Dylan Nagy, Sarah Avina, Daniel Ludwinski, David M. Anderson

**Affiliations:** 1 Department of Health Policy and Management, University of Pittsburgh, Pittsburgh, Pennsylvania; 2 Department of Economics, Oxford College of Emory University, Oxford, Georgia; 3 Department of Health Services, Policy and Management, University of South Carolina, Columbia

## Abstract

**Importance:**

Millions of lower-income Health Insurance Marketplace enrollees were defaulted from zero-premium to positive-premium health plans in 2022, 2023, and 2024. This turnover in zero-premium plans may cause coverage losses by creating administrative burdens that complicate enrollees’ ability to maintain coverage.

**Objective:**

To determine how turnover affected Marketplace reenrollment.

**Design, Setting, and Participants:**

This cross-sectional study used log-linear fixed-effects models including counties in 29 states that used the HealthCare.gov platform from 2022 through 2024.

**Exposure:**

HealthCare.gov enrollees living in a county that experienced turnover that year.

**Main Outcomes and Measures:**

County-year–level counts of overall reenrollment, automatic and active enrollment, and active reenrollment split by whether enrollees stayed with or switched from their previous plan. We controlled for premium affordability, insurer competition, other county characteristics, and state-by-year policy changes.

**Results:**

The sample consisted of 2159 counties representing roughly 10 million HealthCare.gov enrollees annually in 29 states that used the HealthCare.gov platform from 2022 through 2024. The share of enrollees living in counties exposed to turnover increased from 10.3% to 93.9% from 2021 to 2022 as the American Rescue Plan Act subsidies were implemented. These increases have persisted into 2024. Turnover across insurers was associated with a 7.0% (95% CI, −12.7 to −1.3) decrease in automatic reenrollment. Any turnover was not associated with changes in active enrollment, though it was associated with a 13.4% decrease (95% CI, −17.7 to −9.1) in enrollees choosing to stay with their previous, default plan and a roughly equivalent 15.0% increase (95% CI, 11.5-18.5) in enrollees choosing to switch plans.

**Conclusions:**

Turnover affects coverage losses by decreasing automatic, passive reenrollment among lower-income enrollees that may not realize they need to start paying premiums to retain coverage that previously did not have a premium. Turnover also nudges returning enrollees to select new plans rather than selecting their previous plans. This likely increases insurer price competition but also may create hassles for enrollees. These findings suggest that coverage losses from turnover in 2026 among lower-income Marketplace enrollees may be particularly large if enhanced subsidies from the Inflation Reduction Act expire.

## Key Points

**Question:**

Do lower-income Health Insurance Marketplace enrollees lose or change coverage when they experience turnover and are defaulted from a zero-premium silver plan to a silver plan with a premium?

**Findings:**

In this cross-sectional study including 2159 counties representing roughly 10 million HealthCare.gov enrollees annually in 29 states, zero- to positive-premium plan turnover was associated with an approximately 7% decrease in automatic reenrollment; turnover also was associated with a roughly 14% increase in enrollees selecting new plans in lieu of their prior plans.

**Meaning:**

Defaulting lower-income Marketplace enrollees from zero- to positive-premium plans reduced automatic reenrollment and prompted some returning enrollees to select a new plan rather than staying with their prior plan.

## Teaser

This cross-sectional study estimates how zero-premium silver plan turnover affected Marketplace coverage in 29 HealthCare.gov states after the implementation of the American Rescue Plan Act.

## Introduction

A record 24.2 million individuals in the US enrolled in the Health Insurance Marketplaces created by the Patient Protection and Affordable Care Act (ACA) in 2025.^1^ Whether these increases in Marketplace enrollment result in sustained coverage increases, however, will depend on how difficult it is for enrollees to stay insured in the coming years. Millions of Medicaid enrollees, for example, recently lost coverage at the end of the federal Public Health Emergency for COVID-19 because they did not submit paperwork to redetermine their Medicaid eligibility.^2^ In this study, we examine a similar phenomenon in the Marketplaces that may lead to substantial coverage losses, particularly if expanded Marketplace premium subsidies expire in 2026.

The passage of the American Rescue Plan Act (ARPA) and the Inflation Reduction Act (IRA)^29^ made up to 2 zero-dollar premium silver health insurance plans available to most lower-income Marketplace enrollees—those with incomes below 150% of the federal poverty level (FPL), about $23 475 for an individual or $48 225 for a family of 4 in 2025.^3,4,5^ These plans’ low premiums and minimal cost-sharing make them a popular choice among enrollees.^6,7^ Which silver plans have zero-dollar premiums can and often do change each year.^8^ When zero-premium silver plan turnover occurs, enrollees that signed up for a zero-premium silver plan in the prior year must either begin paying a premium or switch to a new zero-premium plan to maintain coverage. A prior study^3^ found that this turnover in zero-premium silver plans occurred for at least 1 silver plan in 93% of counties using the HealthCare.gov platform in 2022.

Turnover in zero-premium silver plans creates 2 administrative burdens to reenrollment.^9^ First, learning costs require that enrollees are aware of their coverage changes and understand how to act in response.^8^ Enrollees seldom pay attention to yearly coverage changes.^10,11,12,13^ Second, compliance costs require that enrollees navigate the hassle of changing their plan or setting up a recurring payment.^9^ This can be particularly difficult for lower-income enrollees, who disproportionately lack access to credit cards and bank accounts.^14^

Two pre-ARPA studies suggest administrative burden substantially reduces reenrollment. One study found that turnover in Massachusetts’ Marketplace caused a 14% coverage decrease among affected lower-income enrollees in 2017.^13^ Another study on California’s Marketplace found that requiring enrollees to pick a new plan to stay covered reduced the probability they maintained coverage by 30 percentage points in 2015 and 2017.^11^ However, the literature has not examined the impact of zero-premium turnover in the states that use the HealthCare.gov platform that covers 76% of Marketplace enrollees nationwide,^15^ nor has it studied this phenomenon since ARPA enhanced subsidies were implemented in mid-2021.

In this study, we estimated how zero-premium silver plan turnover affected Marketplace coverage in 29 HealthCare.gov states after the implementation of ARPA. To do so, we compared reenrollment in counties that had zero-premium silver plans in the prior year by whether they experienced turnover in zero-premium plans in the subsequent year. Our findings have important policy implications for state and federal policymakers seeking to reduce administrative barriers and coverage losses for lower-income populations, which will be especially important should ARPA/IRA subsidies expire, as scheduled, in 2026.

## Methods

We examined how exposure to zero-premium silver plan turnover, overall and across insurers, is associated with HealthCare.gov reenrollment at the county-year level. We use plan offering and reenrollment data from the Centers for Medicare and Medicaid Services (CMS) on states that used the HealthCare.gov platform continuously from 2022 through 2024, following the implementation of expanded ARPA premium subsidies. Our approach compared counties that experienced turnover to those that did not. This cross-sectional study was deemed exempt from review because it used only secondary deidentified data. We followed the Strengthening the Reporting of Observational Studies in Epidemiology (STROBE) reporting guidelines.

### Data

Our primary data were from the Qualified Health Plan (QHP) Landscape File from HealthCare.gov and the Marketplace Open Enrollment Period (OEP) Public Use Files from the Center for Consumer Information and Oversight (CCIIO). The QHP identifies each county-year–level health plan offering on HealthCare.gov, along with premiums and benefits. The OEP Public Use Files record HealthCare.gov county-year–level effectuated reenrollment stratified by type, such as automatic vs active reenrollment, though it does not report reenrollment stratified by income or other demographic characteristics. We also use the CCIIO Plan ID Crosswalk to identify the default plan that enrollees are assigned from 1 year to the next.

### Sample

The sample includes 29 states that used HealthCare.gov in each year from 2022 through 2024. We excluded Alaska and Hawaii because FPL is defined differently in those states, and Nebraska because it does not use county lines to designate where Marketplace plans can be sold. We excluded at most 3% of county-years in our sample from our analyses, depending on the outcome, where enrollment below 11 persons was not reported or where 2023 to 2024 crosswalk data were not provided (eAppendix 1 and eTable 2 in Supplement 1). We used 2021 QHP data to identify turnover from 2021 to 2022, 2021 OEP data to calculate 2021 enrollment weights, and 2019 to 2021 QHP and OEP data to examine pre-ARPA turnover.

### Reenrollment Outcomes

We have 5 county-year–level reenrollment count outcomes. These include: (1) overall reenrollment; (2) automatic or passive reenrollment, whereby returning enrollees take no action and remain covered through their default plan, typically their plan from the previous year; (3) any active reenrollment, whereby returning enrollees remain enrolled by actively selecting a health plan; (4) active reenrollment staying, whereby enrollees choose to keep their previous plan; (5) active reenrollment switching, whereby enrollees choose a different plan. All enrollees covered as of December 31 of the prior year may choose to reenroll passively or actively.

That OEP data report effectuated enrollment on January 15 has important implications for the overall and automatic reenrollment measures. Whether returning enrollees experiencing turnover have their coverage eliminated by January 15 depends not only on whether they make a premium payment, but also whether they are defaulted to a plan offered by their previous insurer or to a new insurer. If reenrollees experience turnover with the same insurer, their coverage cannot be terminated for missing premium payments before January 31. However, if enrollees experience zero- to positive-premium plan turnover to a different insurer, they must make a premium payment by January 1 to retain coverage. Therefore, our data only identify turnover-related coverage losses when enrollees are turned over to new insurers. This limitation does not apply, however, to active reenrollment measures because enrollees must make active plan selections prior to January 15. We thus focused our analysis of overall and automatic reenrollment on county-years experiencing turnover across insurers, and our analysis of active reenrollment on county-years with any turnover.

### Exposure: Turnover in Zero-Premium Plans

We identified county-years where there was zero-premium silver plan turnover. For each county-year, we first calculated enrollees’ postsubsidy premiums for the 2 lowest-premium silver plans in the previous year, and the plans to which those plans defaulted in the current year. If available, a plan defaults to itself. If not, a similar plan becomes the new default. We identified defaults using the plan crosswalk data.^16^ Next, we coded county-years as experiencing turnover if either or both of the lowest-premium and second-lowest premium silver plans (1) had a zero-dollar premium in the previous year; and (2) defaulted to a plan with a positive premium. Turnover is an intent-to-treat measure in that it identifies counties where turnover in zero-premium plans occurred, not the number of enrollees that were affected by turnover. We cannot observe how many enrollees were affected by turnover because the OEP public use files data do not report this information. However, roughly half of lower-income Marketplace enrollees select either the lowest-premium or second-lowest premium silver plan, suggesting many enrollees experience turnover.^17^ We performed these calculations for a single, 40-year-old enrollee with income between 100% to 150% FPL (eAppendix 2 in Supplement 1).

### Statistical Analysis

We used multivariable log-linear fixed-effects regression models to estimate the associations between reenrollment and counties experiencing zero-premium silver plan turnover. We retransformed our estimates, calculating standard errors using the δ method, to interpret estimates as percentage changes in reenrollment. When examining overall and automatic reenrollment, we focused on their association with turnover across insurers because only this type of turnover can result in coverage termination prior to January 15, the date which is reflected in the CMS enrollment data. When examining active enrollment, we focused on its association with any turnover because returning enrollees must make active plan selections prior to January 15. We provide associations between all types of reenrollment and both types of turnover for completeness and as a falsification test. Analysis occurred between July 1, 2024, to March 10, 2025, using R statistical software (R Foundation for Statistical Computing).

We controlled for bronze spreads, a measure of affordability for subsidized Marketplace coverage,^18^ and the number of competing insurers (1, 2, ≥3). Prior Marketplace research has found that premium affordability is essentially the only plan-level characteristic that affects whether people choose to enroll (ie, bronze spreads),^19^ and insurer competition can affect plan offerings.^3,8,18^ We included both county and state-by-year fixed effects. County fixed effects control for time-invariant county characteristics, including sociodemographic and health characteristics. State-by-year fixed effects capture time-varying changes in state policies, notably Medicaid expansion. We weighted counties by their 2021 enrollment and clustered standard errors at the county level. We did not control for demographic characteristics because these characteristics exhibited relatively little variation over the study period and were thus largely captured by county fixed effects (eAppendix 3 in Supplement 1).

Our approach relies on rich fixed effects to isolate the effects of turnover on reenrollment. Because our turnover occured at the county level, we used state-by-year fixed effects to control for all variation in state-level Marketplace policies. Our fixed-effects identification strategy therefore identifies associated effects if there are no unobserved county-year–varying confounders affecting both exposure to turnover and reenrollment.

## Results

### Descriptive Analyses

The sample consists of 6471 county-years representing 36.7 million enrollee-years in 2157 unique counties in 29 states from 2022 to 2024. Of those county-years, 4452 experienced any turnover and 211 experienced turnover across insurers, representing 28.4 and 0.8 million enrollee-years, respectively. Roughly 9.6 million enrollees received Marketplace coverage in these counties in 2022 during open enrollment, which increased to 11.5 million in 2023 and 15.9 million in 2024. As shown in Table 1, approximately 75.2% of total Marketplace enrollment from 2022 through 2024 was enrollees from the previous year reenrolling. Among these reenrollees, roughly 28.6% automatically reenrolled in their previous plan and 71.4% actively selected a plan each year—40.4% staying with their previous plan and 31.0% switching. These percentages were stable over time.

**Table 1.** HealthCare.gov Reenrollment by Year, 2022 to 2024^a^^,^^b^

Variable	Year, % of enrollment			
	2022	2023	2024	Total
Reenrollment type				
Overall	76.7	75.3	74.1	75.2
Automatic	21.2	20.8	22.1	21.5
Active	55.4	54.6	52.0	53.7
Stay with previous plan	24.0	23.2	23.3	23.4
Switch to new plan	31.5	31.4	28.7	30.3
Reenrollment type				
Automatic	27.7	27.6	30.0	28.6
Active	72.3	72.4	70.0	71.4
Stay with previous plan	31.1	30.4	31.3	31.0
Switch to new plan	41.2	42.0	38.7	40.4

^a^ The sample includes counties in states that continuously used HealthCare.gov from 2019 to 2024, excluding Alaska, Hawaii, and Nebraska. All percentages are weighted by county-year enrollment. Automatic reenrollment occurs when returning enrollees do not return to HealthCare.gov and remain enrolled by continuing to pay the premium of their default plan, which may or may not be zero. Active reenrollment occurs when enrollees return to HealthCare.gov to select a plan, either staying with their previous plan or switching to a new plan. Enrollees also may actively select a plan through third-party platforms or brokers.

^b^ The enrollee-weighted means shown in the Figure.

Table 2 reports 2021 enrollment-weighted mean outcomes overall, by whether counties experienced any turnover, by turnover across insurers, and differences between these groups. Active staying was 5.7–percentage points (95% CI, −7.8 to −3.6) lower in county-years experiencing any turnover—relative to those not experiencing it—and active switching was 8.0–percentage points (95% CI, 4.3-11.7) higher. Counties with 3 or more insurers were significantly more likely to experience turnover, which is indicative of price competition changing which plans are the lowest-premium silver plans.

**Table 2.** Income and Demographic Characteristics of Healthcare.Gov Enrollees in Zero-Premium Silver Plans by Any Turnover and Turnover Across Insurers, 2022 to 2024^a^

County-year characteristic	Overall	Turnover	No turnover	Difference (95% CI)
**Any turnover, other counties**				
No.	6459	2007	4452	NA
Reenrollment, %				
Overall	75.8	76.0	75.3	0.5 (−0.6 to 1.5)
Passive	28.6	28.1	30.2	−2.3 (−5.3 to 0.8)
Active	71.4	71.9	69.8	2.3 (−0.8 to 5.3)
Stay	31.8	30.5	35.9	−5.7 (−7.8 to −3.6)
Switch	39.7	41.4	33.9	8.0 (4.3 to 11.7)
Plan offerings, mean (SD)				
Bronze spread, $	117.3 (40.7)	118.0 (40.7)	115.2 (40.4)	0.2 (−7.2 to 7.6)
No. of insurers				
1	2.5	2.1	3.9	−2.3 (−3.6 to −1.0)
2	10.2	10.0	10.6	−2.0 (−5.4 to 1.4)
≥3	87.3	87.9	85.4	4.3 (0 to 8.5)
**Turnover across insurers, other counties**				
No.	6459	211	6248	NA
Reenrollment, %				
Overall	75.8	76.0	75.8	0.6 (−1.0 to 2.2)
Passive	28.6	30.9	28.5	1.9 (−2.7 to 6.6)
Active	71.4	69.1	71.5	−1.9 (−6.6 to 2.7)
Stay	31.8	33.4	31.7	1.4 (−0.5 to 3.3)
Switch	39.7	35.7	39.7	−3.3 (−7.8 to 1.2)
Plan offerings				
Bronze spread, $	117.3 (40.7)	105.1 (18.4)	117.6 (41.0)	−13.9 (−21.6 to −6.2)
No. of insurers				
1	2.5	0	2.6	−2.2 (−3.2 to −1.2)
2	10.2	0.3	10.4	−9.0 (−11.9 to −6.2)
≥3	87.3	99.7	87.1	11.2 (7.6 to 14.8)

Abbreviation: NA, not applicable.

^a^ Counties experiencing turnover have either or both of their lowest-premium and second-lowest premium silver plans turnover from 1 year to the next. Turnover across insurers occurs when enrollees in either of or both of the lowest-premium and second-lowest silver plans are turned over to a plan offered by a different insurer than with whom they were enrolled with in the previous year. All statistics are calculated using 2021 county enrollment weights. The difference column reports the county-clustered, year-adjusted difference between county-years with and without turnover.

The implementation of enhanced ARPA subsidies in mid-2021 made zero-premium silver plans available to many lower-income enrollees for the first time, leading to large increases in zero- to positive-premium silver plan turnover in 2022. Whereas 10.2% of Marketplace enrollees lived in counties with turnover in 2021 before the passage of ARPA, 93.9% did so in 2022, 67.9% did so in 2023, and 83.4% did so in 2024 (Figure). Turnover among enrollees with incomes from 150% to 200% FPL was markedly lower; roughly 2.8% of these enrollees lived in counties experiencing turnover in 2021, and 7.7% did so in 2022 (eFigure in the Supplement 1 for county by year maps of turnover exposure).

**Figure.**
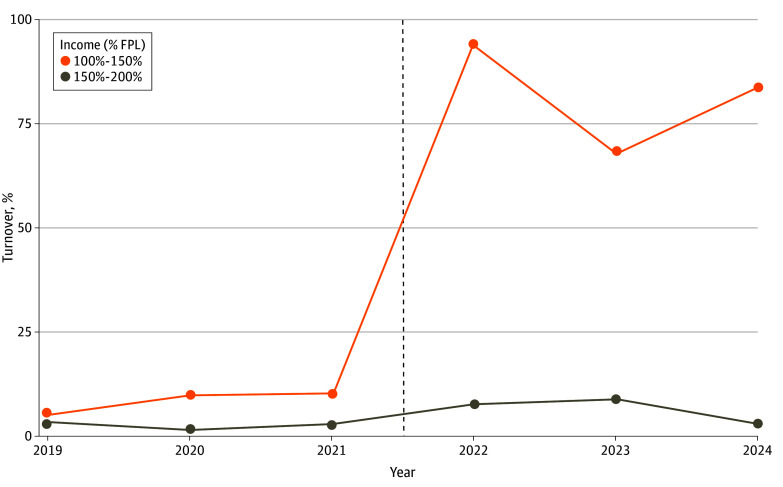
HealthCare.gov Enrollees Living in Counties With Turnover in Zero-Premium Silver Plans, 2019 to 2024^a^ FPL indicates federal poverty line. The dotted line indicates the implementation of enhanced American Rescue Plan Act premium subsidies. ^a^The sample includes counties in states that continuously used healthCare.gov from 2019 to 2024, excluding Alaska, Hawaii, and Nebraska. All percentages are weighted by county-year enrollment.

### Regression Analyses

Turnover was associated with a 13.4% (95% CI, −17.7% to −9.1%) decrease in active staying and a corresponding 15.0% (95% CI, 11.5%-18.5%) increase in active switching in counties experiencing any turnover (Table 3). We did not find that any turnover was associated with overall reenrollment, automatic passive reenrollment, or active reenrollment overall. Increases in bronze spreads were associated with increases in overall reenrollment, active reenrollment, and active switching. Increased insurer competition also was associated with increases in active switching.

**Table 3.** Associations of Zero-Premium Silver Plan Turnover With Reenrollment^a^

Turnover type, covariate	Reenrollment type, coefficient (95% CI)					
	Overall	Passive	Active		Active stay	Active switch
**Any turnover**						
Turnover	0.6 (−1.2 to 2.4)	2.3 (−1.0 to 5.7)		0.2 (−1.4 to 1.9)	−13.4 (−17.7 to −9.1)	15.0 (11.5 to 18.5)
Bronze spread	0.1 (0 to 0.1)	0 (−0 to 0.1)		0.1 (0.1 to 0.1)	0 (−0.2 to 0.1)	0.2 (0.1 to 0.3)
No. of insurers						
1	NA	NA		NA	NA	NA
2	0.1 (−5.8 to 6.0)	−4.6 (−12.6 to 3.4)		2.1 (−3.8 to 8.0)	−7.0 (−19.4 to 5.5)	21.0 (5.7 to 36.3)
3	1.8 (−5.4 to 9.1)	−7.6 (−16.9 to 1.8)		5.7 (−1.7 to 13.1)	−4.8 (−19.7 to 10.1)	27.0 (9.5 to 44.4)
**Turnover across insurers**						
Turnover	−3.6 (−8.6 to 1.4)	−7.0 (−12.7 to −1.3)^b^		−3.4 (−9.7 to 3.0)	−13.2 (−27.6 to 1.3)	0.6 (−16.3 to 17.4)
Bronze spread	0.1 (0 to 0.1)	0 (−0 to 0.1)		0.1 (0.1 to 0.1)	−0.1 (−0.2 to 0.1)	0.2 (0.1 to 0.3)
No. of insurers						
1	NA	NA		NA	NA	NA
2	0.1 (−5.7 to 6.0)	−4.3 (−12.2 to 3.5)		2.1 (−3.8 to 8.0)	−8.7 (−22.0 to 4.6)	23.3 (7.1 to 39.4)
3	1.9 (−5.3 to 9.2)	−7.3 (−16.5 to 1.9)		5.8 (−1.6 to 13.1)	−5.9 (−21.9 to 10.2)	28.5 (10.2 to 46.9)
County-years, No.	6310	6285		6285	6239	6239

Abbreviation: NA, not applicable.

^a^ All models are estimated with county and state-by-year fixed effects with 2021 enrollment weights and county-clustered error terms. Coefficients and standard errors are retransformed, using the delta method, and multiplied by 100 so they may be interpreted as percentage changes in the outcomes.

^b^ *P* < .001.

Turnover across insurers was associated with a 7.0% (95% CI, −12.3% to −1.3%) decrease in passive automatic reenrollment. Turnover across insurers was not associated with changes in other types of reenrollment. To test the robustness of the passive reenrollment finding, we estimated an alternate model where we compared county-years experiencing turnover across insurers to those experiencing any turnover, limiting the sample to counties with similar insurance market dynamics. Here, turnover across insurers was associated with an 8.4–percentage point (95% CI, −14.1 to −2.8) decrease in passive reenrollment (eTable 2 in Supplement 1).

## Discussion

To our knowledge, this cross-sectional study provides the first post-ARPA evidence on the association of zero-premium silver plan turnover effects with HealthCare.gov–based Marketplace reenrollment. Enrollees experiencing turnover lose Marketplace coverage, potentially becoming uninsured, unless they set up a monthly premium payment for their health plan or switch to a new zero-premium plan. Among enrollees who would lose coverage by January 1—those defaulted to a new insurer—we found turnover affected a 7% decrease in automatic reenrollment independently of changes in premiums; we did not find offsetting increases in other types of reenrollment. Given that enrollees reenrolling automatically comprised 21.5% of HealthCare.gov enrollees in 2024, our findings suggest that turnover may have reduced total 2024 HealthCare.gov reenrollment by about 1.5%, or approximately 250 000 people.

We also found that, although turnover was not associated with overall changes in returning enrollees actively selecting their plan, it was associated with a 15–percentage point increase in reenrollees actively switching their plans and a roughly offsetting 13–percentage point decrease in reenrollees actively choosing to stay with the same plan. Independently of changes in premium affordability, these findings suggest that returning, lower-income enrollees may be more likely to switch plans when they are defaulted away from zero-dollar silver plan coverage. Lower-income enrollees, who often have limited access to credit cards and bank accounts,^21^ may value avoiding the hassle costs of making a monthly premium payment to their insurer and switch to new zero-premium plans accordingly.

These findings are broadly consistent with prior studies examining turnover in Massachusetts and California.^11,13^ Our analysis generalized these findings to HealthCare.gov, the model platform for Marketplace coverage, as well as the post-ARPA and IRA subsidy regime.^22^ Collectively, this expanding body of work continues to demonstrate that the administrative burdens of paying small premiums poses considerable barriers to continuity of coverage for Marketplace enrollees, particularly those with lower incomes. Enhanced Marketplace subsidies are scheduled to expire in 2026. The Congressional Budget Office projected that subsidy reductions will reduce Marketplace enrollment by 2.2 million enrollees, about 9% of total Marketplace enrollment.^23^ It is unclear if these projections account for coverage losses due to administrative burdens. Zero-dollar silver plans will largely cease to exist without enhanced subsidies, so zero- to positive-premium silver plan turnover will affect lower-income Marketplace enrollees nationwide unless enhanced subsidies are extended.^20^

That we can eliminate all state-year policy variation as a potential source of bias is a key strength of this study. Differential county-year-level pricing and plan offering behavior affecting turnover exposure and reenrollment remains as a potential source of bias; however, insurers often pursue market strategies at the state-year level rather than the county-year level, suggesting that our state-year fixed effects may be sufficient for association identification.

Our approach has trade-offs relative to commonly used difference-in-differences (DD) approaches. Standard DD approaches typically cannot control for state-year policy variation as we do because they rely on state-year variation to identify state policy changes. However, our approach lacks a comparison to the pretreatment period. We did not pursue a DD approach because the preperiod—the Marketplaces without ARPA/IRA subsidies—was fundamentally different for lower-income enrollees in terms of zero-premium plan availability.^20^

State policy makers could mitigate turnover-related coverage losses by implementing automatic retention policies similar to the pre-ACA Massachusetts Marketplace, whereby enrollees that ceased premium payments were defaulted to a zero-dollar premium plan.^24^ Although the effectiveness of automatic retention would be reduced without ARPA- and/or IRA-enhanced subsidies, states could use targeted wraparound subsidies to retain zero-dollar premium silver plan availability for some lower-income enrollees. Ten states currently have some form of wraparound subsidy.^25^

The effects of turnover are not uniformly negative. Turnover nudges enrollees to select a new health plan. Prior literature consistently finds that people pick health plans that better align with their preferences when they have to select a plan.^26,27^ When enrollees actively review their plan options, it incentivizes insurers to compete by lowering premiums or improving benefits.^10,28^ Yet turnover also imposes administrative burdens on enrollees that may have monetary, time, and/or psychological costs.^9^ Further research is therefore needed to better understand the costs and benefits of turnover for enrollees that return in spite of its costs, though the effects of turnover are clearly negative for those that lose coverage.

### Limitations

We recognize that this cross-sectional study has the following limitations. First, CCIIO OEP data limit the analysis of the effects of turnover on overall and passive reenrollment to counties experiencing turnover across insurers, rather than any county experiencing turnover. Second, our analysis was limited to 29 states using the HealthCare.gov platform. Our findings, however, are consistent with prior evidence from state-based Marketplaces.^11,13^ Third, we estimated intent-to-treat effects rather than average treatment effects on the treated because we did not observe how many enrollees were affected by turnover. These estimates still allowed us to estimate the effects of turnover, though these estimates are less precise.

## Conclusions

We examined how zero-premium silver plan turnover is associated with Marketplace enrollment on the federally-facilitated Marketplace. We found that turnover was associated with reduced automatic reenrollment by roughly 7%, and that it nudged some returning enrollees to select a new plan rather than opting to stay in their previous one. Expiration of the IRA subsidies will likely lead to coverage losses due to price increases; our findings suggest further coverage losses may occur for lower-income enrollees as a result of the administrative burdens created by turnover.

## Notes

Supplement 1. eFigure 1. Geographic Exposure to Turnover, 2022-2024 eTable 1. Healthcare.Gov Enrollees Living in Counties with Turnover in Zero-Premium Silver Plans, 2019-2024 eTable 2. Associations of Reenrollment in Counties with Zero-Premium Silver Plan Turnover across Insurers, Relative to Counties with Zero-Premium Silver Plan Turnover within Insurers eAppendix 1. Missing Data eTable 3. Counties Excluded from Analytic Sample eAppendix 2. Calculating Premiums And Subsidies eAppendix 3. Regression Analyses

Supplement 2. Data Sharing Statement
