# Supplementary material for: Coverage Retention and Plan Switching Following Switches From a Zero- to a Positive-Premium Plan
Source: JAMA Health Forum. 2025 May 23;6(5):e251424. doi: 10.1001/jamahealthforum.2025.1424 (PMC12102699; doi:10.1001/jamahealthforum.2025.1424)
Supplement: Supplement 1. — eFigure 1. Geographic Exposure to Turnover, 2022-2024 eTable 1. Healthcare.Gov Enrollees Living in Counties with Turnover in Zero-Premium Silver Plans, 2019-2024 eTable 2. Associations of Reenrollment in Counties with Zero-Premium Silver Plan Turnover across Insurers, Relative to Counties with Zero-Premium Silver Plan Turnover within Insurers eAppendix 1. Missing Data eTable 3. Counties Excluded from Analytic Sample eAppendix 2. Calculating Premiums And Subsidies eAppendix 3. Regression Analyses [file jamahealthforum-e251424-s001.pdf]

Supplemental Online Content

Drake C, Nagy D, Avina S, Ludwinski D, Anderson DM. Coverage Retention and Plan Switching Following Switches From a Zero- to a Positive-Premium Plan. *JAMA Health Forum.* Published online May 23, 2025. doi:10.1001/jamahealthforum.2025.1424

eFigure 1. Geographic Exposure to Turnover, 2022-2024
eTable 1. Healthcare.Gov Enrollees Living in Counties with Turnover in Zero-Premium Silver Plans, 2019-2024
eTable 2. Associations of Reenrollment in Counties with Zero-Premium Silver Plan Turnover across Insurers, Relative to Counties with Zero-Premium Silver Plan Turnover within Insurers
eAppendix 1. Missing Data
eTable 2. Counties Excluded from Analytic Sample
eAppendix 2. Calculating Premiums And Subsidies
eAppendix 3. Regression Analyses

This supplemental material has been provided by the authors to give readers additional information about their work.

© 2025 Drake C et al. *JAMA Health Forum*.

eFigure 1. Geographic Exposure to Turnover, 2022-2024[a]

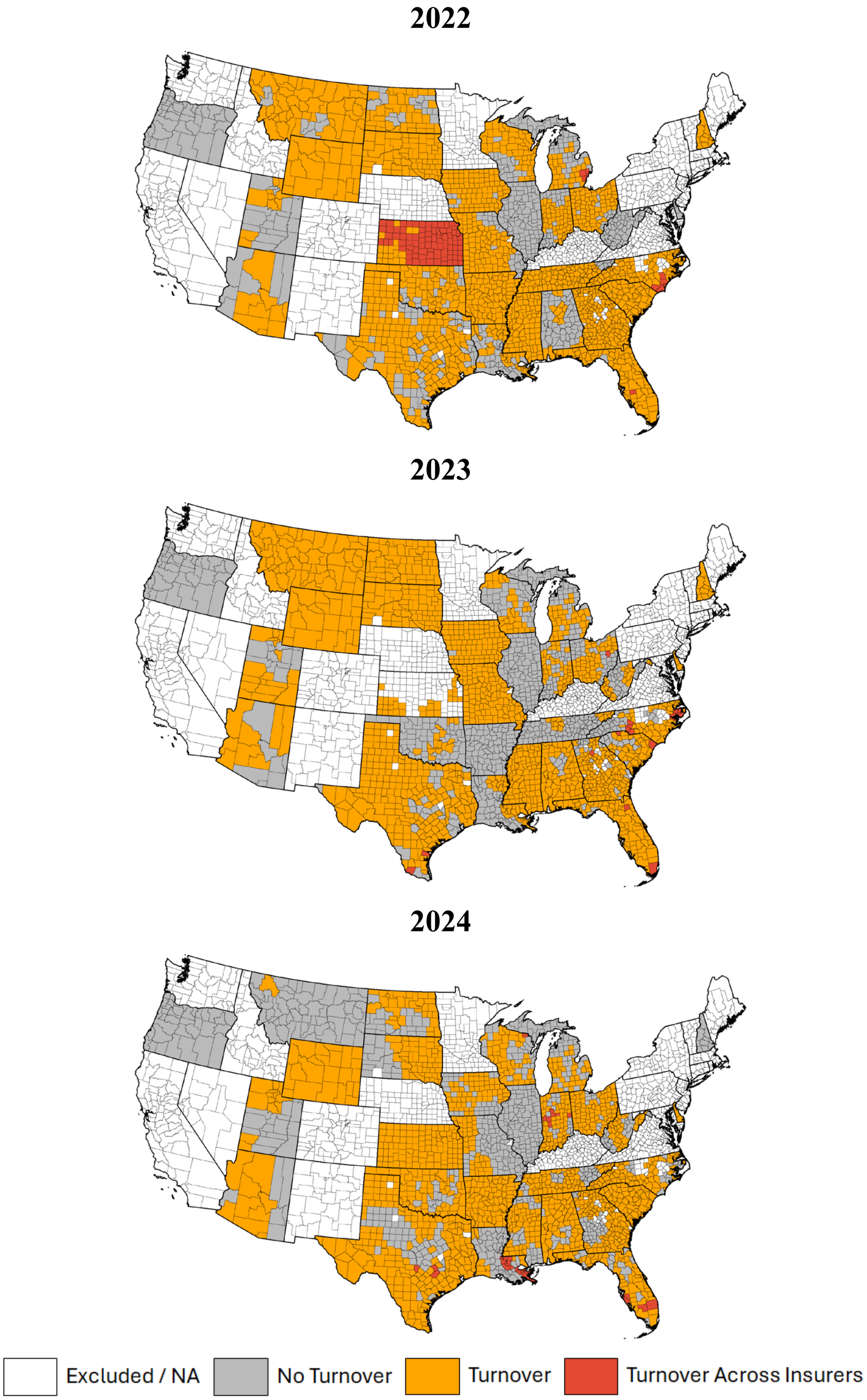

[a] Counties experiencing turnover have either or both of their lowest and second-lowest premium silver plans turnover from one year to the next. Excluded states did not use HealthCare.gov from 2021-2024 or were excluded from the sample (see methods). Excluded counties had missing data (see appendix).

© 2025 Coleman D et al. *JAMA Health Forum*.

eTable 1. Healthcare.Gov Enrollees Living in Counties with Turnover in Zero-Premium Silver Plans, 2019-2024

Income (% FPL)	Year					
	**2019**	**2020**	**2021**	**2022**	**2023**	**2024**
100-150	5.4	10.3	10.3	93.9	67.8	83.8
150-200	3.3	1.6	2.8	7.8	9.2	3.5

© 2025 Coleman D et al. *JAMA Health Forum*.

eTable 2. Associations of Reenrollment in Counties with Zero-Premium Silver Plan Turnover across Insurers, Relative to Counties with Zero-Premium Silver Plan Turnover within Insurers[a]

Turnover Type, Covariate	Reenrollment Type, Coefficient (95% Confidence Interval)				
	**Overall**	**Passive**	**Active**	**Active Stay**	**Active Switch**
**B. Turnover Across Insurers**					
Turnover	-4.5<br>[-9.1, 0.1]	-8.4**<br>[-14.1, -2.8]	-4.2<br>[-10.3, 1.9]	-13.3<br>[-29.0, 2.4]	-2.1<br>[-18.8, 14.6]
Bronze Spread	0.1***<br>[0.0, 0.1]	0.0<br>[-0.0, 0.1]	0.1***<br>[0.1, 0.1]	0.1<br>[-0.2, 0.3]	0.1*<br>[0.0, 0.3]
Insurers (#)					
1	-	-	-	-	-
2	0.5<br>[-5.7, 6.7]	-5.2<br>[-13.9, 3.4]	2.9<br>[-3.2, 9.0]	-1.8<br>[-19.0, 15.4]	23.5*<br>[2.4, 44.6]
3	2.9<br>[-4.7, 10.4]	-7.4<br>[-17.0, 2.3]	6.8<br>[-0.8, 14.4]	4.1<br>[-15.7, 23.9]	27.5*<br>[4.0, 51.1]
**County-Years (N)**	4,100	4,100	4,100	4,100	4,100

[a] All models are estimated with county and state-by-year fixed effects with 2021 enrollment weights and county-clustered error terms. Coefficients and standard errors, using the delta method, are retransformed and multiplied by 100 so they may be interpreted as percentage changes in the outcomes.

© 2025 Coleman D et al. *JAMA Health Forum*.

eAppendix 1. MISSING DATA

Our data include all county-years in states that used the healthcare.gov platform continuously from 2022 through 2024. We restrict our sample as follows:

1. We exclude states that did not use healthcare.gov at any point during the study period from 2022-2024. This excludes KY, ME, NJ, NV, NM, PA, and VA.

2. We exclude states that used different FPL definitions and/or did not use counties to construct markets for their Marketplaces. This excludes AK, HI, and NE.

3. The CCIIO OEP PUF data do not report enrollment when enrollment of a given type (e.g., enrollment in silver CSR 94 plans) is above zero and under 11. This creates variation in our sample size across outcomes.

4. We exclude counties where plans were defaulted across years by ZIP code rather than county, where FIPS codes changed over time, and where plan defaults were not consistent between the QHP and crosswalk data. We list these counties below.

© 2025 Coleman D et al. *JAMA Health Forum*.

eTable 3. Counties Excluded from Analytic Sample

County	FIPS Code	Reason
Shannon County, SD	46113	Changed to 46102 in 2014.
Bedford City, VA	51515	Independent City from 1968 through 2013. Combined with Bedford County on July 1, 2013.
Bedford County, VA	51019	Combined with Bedford City on July 1, 2013.
Baldwin County, GA	13009	No crosswalk data from 2023-2024
Barrow County, GA	13013	No crosswalk data from 2023-2024
Bibb County, GA	13021	No crosswalk data from 2023-2024
Chatham County, GA	13051	No crosswalk data from 2023-2024
Clarke County, GA	13059	No crosswalk data from 2023-2024
Hancock County, GA	13141	No crosswalk data from 2023-2024
Jackson County, GA	13157	No crosswalk data from 2023-2024
Monroe County, GA	13207	No crosswalk data from 2023-2024
Muscogee County, GA	13215	No crosswalk data from 2023-2024
Oconee County, GA	13219	No crosswalk data from 2023-2024
Peach County, GA	13225	No crosswalk data from 2023-2024
Pickens County, GA	13227	No crosswalk data from 2023-2024
Randolph County, GA	13245	No crosswalk data from 2023-2024
Wilkinson County, GA	13319	No crosswalk data from 2023-2024
Alamance County, NC	37001	No crosswalk data from 2023-2024
Caswell County, NC	37033	No crosswalk data from 2023-2024
Chatham County, NC	37037	No crosswalk data from 2023-2024
Edgecombe County, NC	37065	No crosswalk data from 2023-2024
Greene County, NC	37079	No crosswalk data from 2023-2024
Guilford County, NC	37081	No crosswalk data from 2023-2024
Lee County, NC	37105	No crosswalk data from 2023-2024
Nash County, NC	37127	No crosswalk data from 2023-2024
Person County, NC	37145	No crosswalk data from 2023-2024
Pitt County, NC	37147	No crosswalk data from 2023-2024
Randolph County, NC	37151	No crosswalk data from 2023-2024
Rockingham County, NC	37157	No crosswalk data from 2023-2024
Watauga County, NC	37189	No crosswalk data from 2023-2024
Wayne County, NC	37191	No crosswalk data from 2023-2024
Wilson County, NC	37195	No crosswalk data from 2023-2024

© 2025 Coleman D et al. *JAMA Health Forum*.

eAppendix 2. CALCULATING PREMIUMS AND SUBSIDIES

We perform the calculations described under "Exposure: Turnover in Zero-Premium Plans" for a single, 40-year-old enrollee with income between 100-150% FPL. Using such a "representative" enrollee is necessary because our aggregate data do not jointly report characteristics that determine enrollees' subsidies: county, income, age, and household size. We assumed enrollees' incomes at 125% FPL, the midpoint of 100-150% FPL, the income range of the overwhelming majority of enrollees affected by turnover. Neither of these choices should have any bearing on our outcomes, which we confirm in robustness checks, because all subsidized enrollees in the 100-150% FPL income range receive sufficient subsidies to purchase zero-dollar silver plans under ARPA regardless of age. This approach follows prior research listed below using the same Healthcare.gov data.

1. Drake C, Anderson DM. Terminating Cost-Sharing Reduction Subsidy Payments: The Impact Of Marketplace Zero-Dollar Premium Plans On Enrollment. *Health Aff (Millwood)*. 2020;39(1):41-49. doi:10.1377/hlthaff.2019.00345

2. Anderson DM, Golberstein E, Drake C. Georgia's Reinsurance Waiver Associated With Decreased Premium Affordability And Enrollment. *Health Aff (Millwood)*. 2024;43(3):398-407. doi:10.1377/hlthaff.2023.00971

3. Anderson D, Abraham JM, Drake C. Rural-Urban Differences In Individual-Market Health Plan Affordability After Subsidy Payment Cuts. *Health Aff (Millwood)*. 2019;38(12):2032-2040. doi:10.1377/hlthaff.2019.00917

© 2025 Coleman D et al. *JAMA Health Forum*.

eAppendix 3. REGRESSION ANALYSES

We estimated county-year-level enrollment and reenrollment's association with turnover of zero-dollar silver plans. Typically, a plan defaults to itself from one year to the next, though there will be another default if a plan exits a county. We accounted for these defaults using CCIIO's Plan ID Crosswalk.

We calculate plan premiums and subsidies for a representative single, 40-year-old enrollee with an income equal to 125% of the Federal Poverty Level (FPL). We use 125% for all outcomes because turnover's effects should be largely limited to 100-150% FPL (i.e., subsidies are not large enough to provide many enrollees with zero-premium silver plans if they have incomes north of 150% FPL).

We consider whether plans covered non-Essential Health Benefits (non-EHBs) in our calculations. Coverage of non-EHBs precludes a plan from having a zero-dollar premium because federal premium subsidies cannot reduce the cost of covering non-EHBs. Required coverage of non-EHBs in a minority of states including Illinois and Oregon precludes them from having zero-premium plans.

We estimated county-year-level enrollment and reenrollment for each county from 2022 to 2024. For county c in state s in year t, we estimated log enrollment Enr_{cst} at a given income level FPL such that

$$\log(Enr_{cst}) = \alpha + \beta Turn_{cst} + \delta Spread_{cst} + \theta_c + \Theta_{st} + \epsilon_{cst},$$

where $Turn_{cst}$ indicates that the lowest and/or the second-lowest premium silver plan had a zero-dollar premium in county c in state s in the prior year $t-1$, *and* that the same plan is no longer a zero-premium plan in the current year t (i.e., its premium increased from $t-1$ to t). The control variable $Spread_{cst}$ measures the bronze spread—the premium difference between the benchmark plan and the lowest-premium bronze plan. Bronze spreads measure affordability. As they increase, the minimum cost of Marketplace coverage decreases. θ_c and Θ_{st} are county and state-year fixed effects, where the former captures time-invariant county characteristics over the study period (2022-24) and the latter captures state policies, both those that do and do not vary over time (e.g., Medicaid expansion, reinsurance). We cluster standard errors at the county level and weight counties by total county Marketplace enrollment in 2021.

© 2025 Coleman D et al. *JAMA Health Forum*.
