# Supplementary material for: Coverage Retention and Plan Switching Following Switches From a Zero- to a Positive-Premium Plan
Source: JAMA Health Forum. 2025 May 23;6(5):e251424. doi: 10.1001/jamahealthforum.2025.1424 (PMC12102699; doi:10.1001/jamahealthforum.2025.1424)
Supplement: Supplement 2. — Data Sharing Statement [file jamahealthforum-e251424-s002.pdf]

Data Sharing Statement

Data
Data available: Yes
Data types: Other (please specify)
Additional Information: Data are publicly distributed by CMS.
How to access data: Data are publicly distributed by CMS.
When available: With publication

Supporting Documents
Document types: None

Additional Information
Who can access the data: Publicly available.
Types of analyses: They are publicly available.
Mechanisms of data availability: They are publicly available.
Any additional restrictions: They are publicly available.
